# Quantitative Evaluation of Osteocyte Morphology and Bone Anisotropic Extracellular Matrix in Rat Femur

**DOI:** 10.1007/s00223-021-00852-1

**Published:** 2021-05-19

**Authors:** Takuya Ishimoto, Keita Kawahara, Aira Matsugaki, Hiroshi Kamioka, Takayoshi Nakano

**Affiliations:** 1 Division of Materials and Manufacturing Science, Graduate School of Engineering, Osaka University, 2-1 Yamada-Oka, Suita, Osaka 565-0871 Japan; 2 Department of Orthodontics, Graduate School of Medicine, Dentistry and Pharmaceutical Sciences, Okayama University, 2-5-1 Shikata-cho, Kita-ku, Okayama, 700-8558 Japan

**Keywords:** Osteocytes, Lacno-canalicular system, Apatite orientation, Bone quality, Anisotropy

## Abstract

Osteocytes are believed to play a crucial role in mechanosensation and mechanotransduction which are important for maintenance of mechanical integrity of bone. Recent investigations have revealed that the preferential orientation of bone extracellular matrix (ECM) mainly composed of collagen fibers and apatite crystallites is one of the important determinants of bone mechanical integrity. However, the relationship between osteocytes and ECM orientation remains unclear. In this study, the association between ECM orientation and anisotropy in the osteocyte lacuno-canalicular system, which is thought to be optimized along with the mechanical stimuli, was investigated using male rat femur. The degree of ECM orientation along the femur longitudinal axis was significantly and positively correlated with the anisotropic features of the osteocyte lacunae and canaliculi. At the femur middiaphysis, there are the osteocytes with lacunae that highly aligned along the bone long axis (principal stress direction) and canaliculi that preferentially extended perpendicular to the bone long axis, and the highest degree of apatite *c*-axis orientation along the bone long axis was shown. Based on these data, we propose a model in which osteocytes can change their lacuno-canalicular architecture depending on the mechanical environment so that they can become more susceptible to mechanical stimuli via fluid flow in the canalicular channel.

## Introduction

Osteocytes inhabit the bone extracellular matrix (ECM) and are believed to play a pivotal role in mechanosensing and mechanotransduction [1–4] which ultimately result in well mechanically functionalized bone material under a specific mechanical environment. To date, the mechanisms by which osteocytes respond to mechanical stimulation to cause bone mechanical integration and resistance to breaking have been widely investigated using in vitro and in vivo assays at the genetic, cellular, and tissue level [5–12]. However, the major focus of these studies in bone adaptive responses was bone mass and/or BMD.

Recently, ECM micro-organization, and in particular, the anisotropic arrangement of collagen molecules and apatite crystallites, has been recognized, in some situations, as an important determinant factor of bone mechanical function [13, 14], rather than BMD [15]. Although bones are required to mechanically function under the anisotropic stress field, BMD which is defined as a mineral amount per volume [mg/cm^3^] does not encompass any anisotropic features of bone materials and cannot be an explanatory parameter for the anisotropic mechanical functions of bone. It is generally accepted that crystallographic texture and orientation—the orderly arrangements of elemental atoms, ions, and molecules—strongly determine the function of materials, such as metals, ceramics, polymers, etc., independent of their volumetric densities. Bone has unique ECM orientations depending on the bone type [16]; for example, unidirectional orientation is seen in the long bones and mandibular corpus, and two-dimensional orientation is seen in the skull bone, although they have a similar BMD. The bone with a highly oriented ECM showed significant anisotropy in Young’s modulus [17, 18], yield stress [17], ultimate stress [17, 19], and toughness [19, 20], so that the values in the oriented direction were higher, while bone with poorly oriented ECM, such as regenerating bones [21, 22], showed relatively isotropic and degraded Young’s modulus [23]. This represents the importance of appropriately oriented ECM for the mechanical performance of bone.

To date, the osteocyte’s role in regulating bone ECM orientation, which largely determines bone mechanical integrity, is unclear, although the knowledge of the osteocyte’s role in regulating bone mass and BMD is increasing. Vatsa et al. [24] suggested that the osteocyte has the potential to detect the “direction” of principal mechanical loading by observing paxillin that localizes to the mechanosensing site in the cell [25]. Moreover, the structure and orientation of the cellular cytoskeleton are reported to be influenced by external mechanical stimuli, especially, the direction of principal strain [26, 27], which affects cell shape [24, 26]. It is, therefore, hypothesized that the osteocyte changes its cell shape and cytoskeleton organization to effectively detect different mechanical environments [28], which is anisotropic in many cases. These studies, however, did not consider whether and how the anisotropic mechanical environment detected links to ECM orientation in bone.

In this study, we investigated the quantitative correlation between the orientation (anisotropic arrangement) of ECM and anisotropic osteocyte morphology using a rat femur which is mainly uniaxially loaded along bone long axis [29], focusing on the efficacy of the osteocyte in sensing an anisotropic stress field. As a rat model has been widely used to study osteocyte’s mechaobiology, usage of rat model is suitable for the present purpose.

## Materials and Methods

### Animals and Bone Specimen Preparation

Five-week-old male Sprague–Dawley rats (*n* = 7) were purchased from Japan SLC. To eliminate the effects of variations in body weight due to sex differences, only male animals were used in this study. The left femur was harvested after euthanasia. All animal procedures and protocols were approved by the Animal Experiment Committee of Osaka University Graduate School of Engineering (Approval Number: 19-4-2). Longitudinal cortical bone sections (sagittal plane, 200–400 μm) were cut with a diamond band saw (BS-300CP; Exakt Apparatebau, Germany). The sectioned specimens were immediately placed into a fixative for electron microscopy (0.5% glutaraldehyde, 2% paraformaldehyde in 0.05 M cacodylate-sodium buffer, pH 7.4) [30] for 48 h at room temperature. The cortical sections were, thereafter, ground with emery paper (2000 grid; Riken, Japan) to a final thickness of approximately 70 μm and dehydrated progressively in 75%, 95%, and 100% ethanol for 5 min for each step.

### Osteocyte Observation Using Confocal Laser Scanning Microscope

Osteocytes embedded in the bone matrix were visualized via fluorescent staining [31]. FITC (F7250; Sigma-Aldrich, USA), diluted in 100% ethanol at a concentration of 1%, was used to stain the osteocyte lacuna and canaliculus. The specimens were immersed into the solution for 2 h to transfuse fluorescent dye into the lacuna and canalicular space. The cortical specimens were then rinsed in 100% ethanol, air dried, and cover-slipped with a fluorescent mounting medium (S3023; Dako North America, CA, USA).

**Fig. 1:**
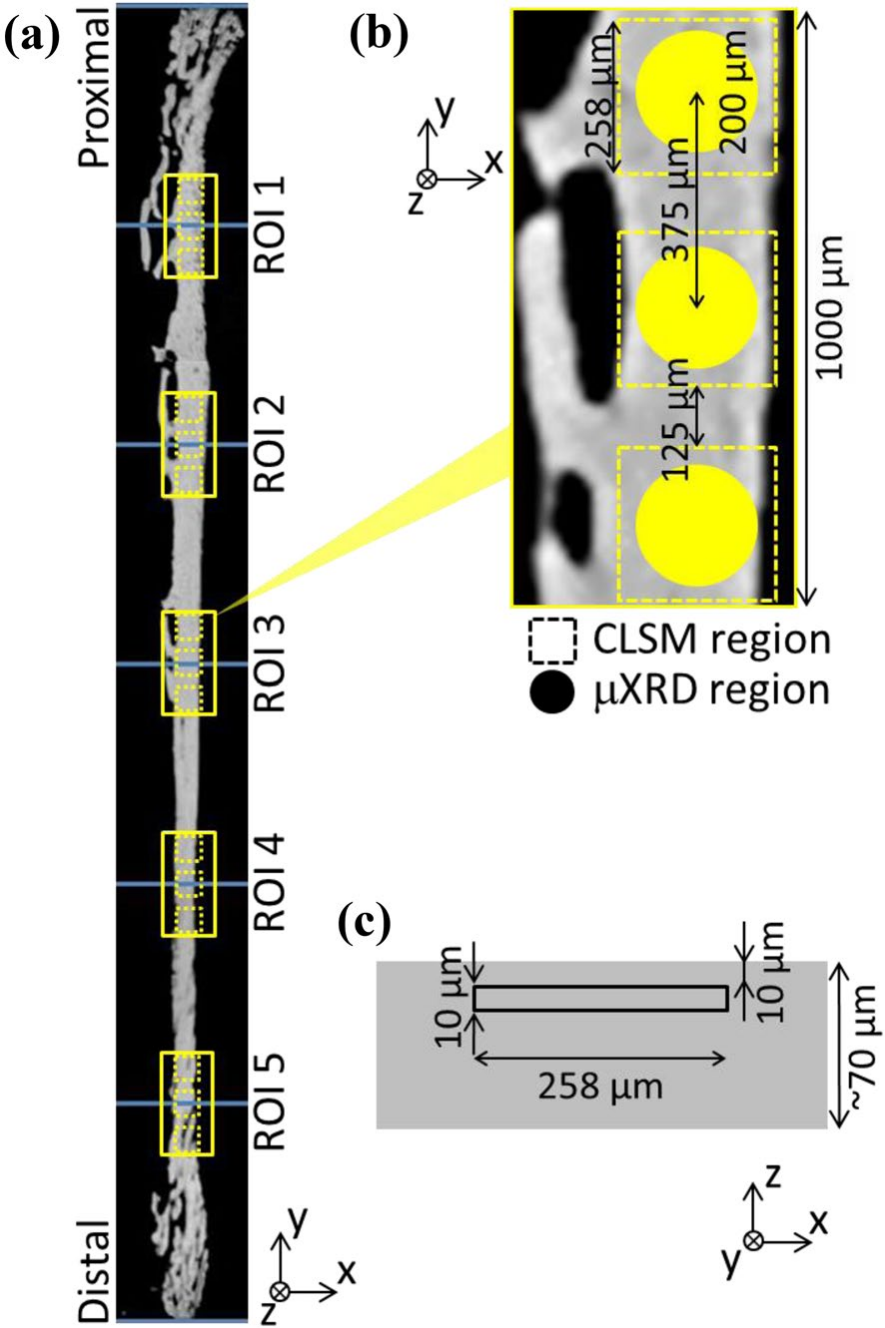
Representations of analyzed portions in rat femur anterior cortices. **a** Five regions of interest (ROIs) along the femur longitudinal axis. **b** Three measurements of microbeam X-ray diffraction and confocal laser scanning microscopy (CLSM) in each ROI were performed. **c** CLSM images were taken from the region indicated by a square (10–20 μm deep from specimen surface)

A confocal laser scanning microscope (CLSM) system (FV1000D-IX81; Olympus, Japan) with UPlanSApo 60 × oil objective lens (numerical aperture = 1.35) was used for osteocyte observation. FITC was excited with a 488-nm laser, and emission fluorescence was detected at a wavelength of 510 nm. The refractive index of the immersion media (Immersion oil Type-F; Olympus, Japan) was 1.518. The theoretical resolutions of the xy-axes and z-axis were 0.184 and 0.565 μm, respectively. The CLSM observations were conducted at the same region of the μXRD measurements (Fig. 1a and b). The frame size of the image (x–y) was 258 μm × 258 μm (0.5 μm/pixel) with a 16-bit color depth. Confocal images were obtained at a depth of up to 10–20 μm from the specimen surface (Fig. 1c), with a 0.5-μm step size (z), which is comparable to the z-axis resolution. In total, 21 images/frames were obtained.

### Quantitative Analysis of Shape and Alignment of Osteocyte Lacunae and Canaliculi

The brightness (*B*) of images taken by CLSM was attenuated exponentially by increasing the depth (*d*) from the specimen surface in which the image was taken; the brightness was adjusted by an exponential function [*B* = *α* exp(−*βd*), where *α* and *β* are fitting parameters] utilizing TRI/3D-BON (RATOC System Engineering, Japan). The images were then binalized and the osteocyte lacunae and canaliculi were separately extracted. Large fluorescent-stained pores corresponding to blood vessels were eliminated from the analyses. The osteocyte lacunae and canaliculi were projected onto the x–y plane and the projected 2D image was used for analyses.

All quantitative analyses of osteocyte lacunae and canaliculi were performed using CellProfiler (www.cellprofiler.org) [32]. Osteocyte morphology was individually approximated to an ellipse which has an equivalent cross-sectional moment of inertia as the original osteocyte lacuna. The aspect ratio (*AR*) of the osteocyte lacuna was calculated as

$$AR ={L}_{\mathrm{L}}/{L}_{\mathrm{S}},$$

where *L*_L_ and *L*_S_ are the long and short axis length of the elliptically approximated osteocyte lacuna, respectively. The degree of osteocyte lacuna alignment (*DA*) along a reference axis was quantitated using the following equation [33]

$$DA=2\left(\langle {\mathrm{cos}}^{2}\varphi \rangle -0.5\right)$$

$$\langle {\mathrm{cos}}^{2}\varphi \rangle ={\int }_{0}^{2\uppi }{\mathrm{cos}}^{2}\varphi \bullet n\left(\varphi \right)d\varphi /{\int }_{0}^{2\uppi }n\left(\varphi \right)d\varphi ,$$

where *ϕ* is an angle between the osteocyte long axis and the longitudinal axis of the femur as a reference axis (y-axis), and < cos^2^*ϕ* > is the averaged cos^2^*ϕ* for all osteocyte lacunae within the analyzed area. If the osteocyte lacunae in the analyzed region are completely aligned parallel and perpendicular to the bone longitudinal axis, *DA* should be 1 and − 1, respectively. Random osteocyte lacunar alignment was assigned a *DA* of 0. Osteocyte canaliculi directionality was also quantified as the angle of canaliculi at their base with respect to the long axis of the lacuna of the canaliculi.

### Analysis of the Preferential Orientation of Apatite *c*-Axis

**Fig. 2:**
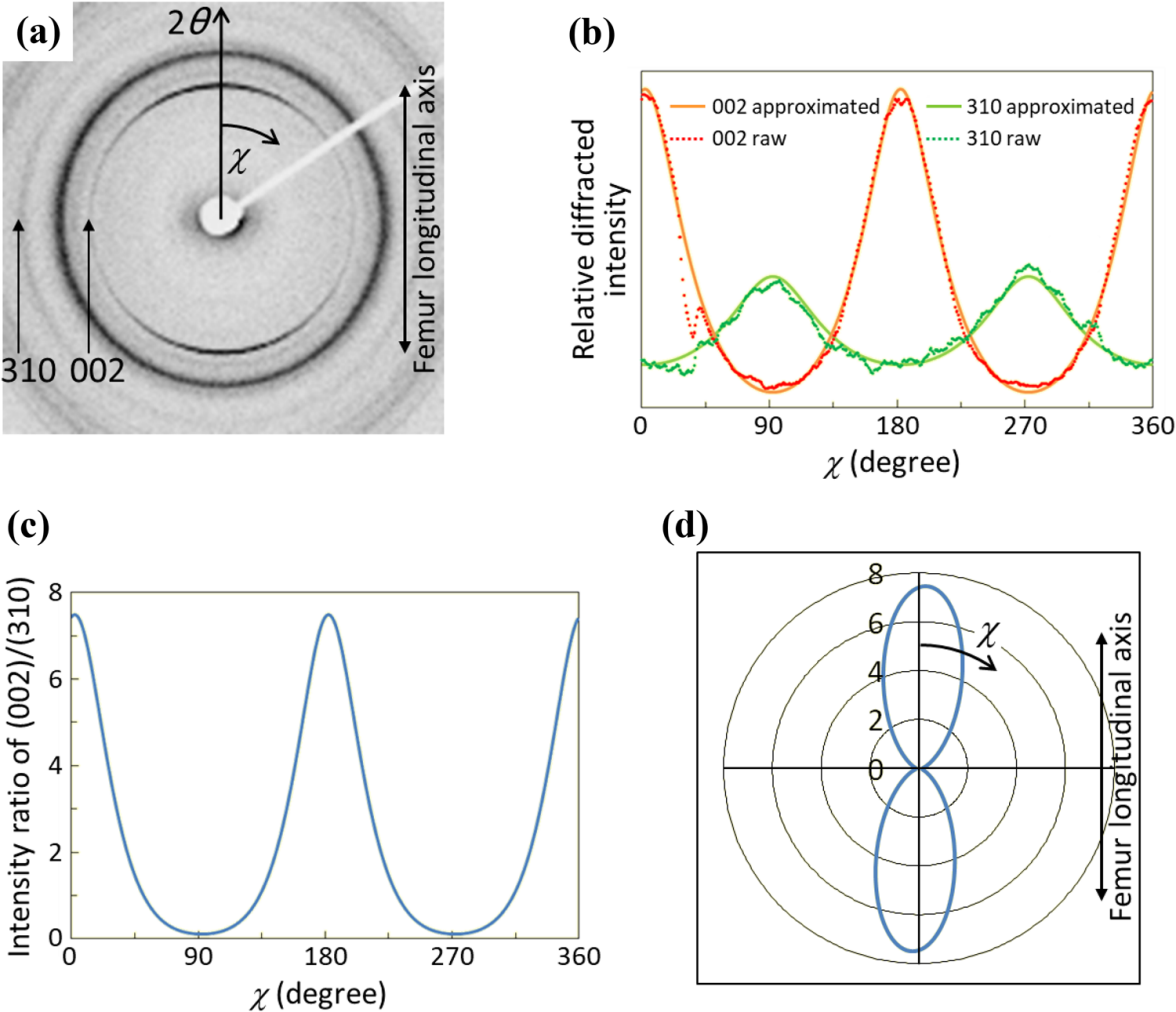
Quantitative analysis of the degree of preferential orientation of apatite *c*-axis along bone long axis. **a** Typical microbeam X-ray diffraction pattern obtained from thinned rat femur. Vertical direction in the image corresponds to femur long axis. **b** Variation in diffraction intensity of (002) and (310) plane of apatite as a function of azimuthal angle *χ.* **c** Distribution of the degree of preferential apatite *c*-axis orientation defined as the intensity ratio of (002) and (310) diffraction peak as a function of azimuthal angle *χ.* **d** Two-dimensional radar diagram of the apatite orientation. The radius represents the intensity ratio of (002)/(310)

In bone tissues, apatite crystallizes on the collagen template in an epitaxial manner [34] through an in vivo self-assembly process. As a result, the crystallographic *c*-axis of apatite is almost parallel to the longitudinal direction of the collagen fibrils [35]. Therefore, the preferential apatite *c*-axis orientation mirrors collagen orientation. The degree of preferential apatite *c*-axis orientation was analyzed conducting a microbeam X-ray diffractometer (μXRD) (R-Axis BQ; Rigaku, Japan) with a transmission optical system and Mo target. A Mo-Kα line was generated at 50 kV and 90 mA (4.5 kW) and a double-pinhole metal collimator with a diameter of 200 μm was used. The μXRD measurements were carried out in the five regions of interest (ROI 1–5) in the anterior cortical bone as shown in Fig. 1a; ROI 3 corresponds to the middiaphysis of the femur. In each ROI, three observations were carried out as described in Fig. 1b and the data were averaged. Incident beam was radiated perpendicularly to the thinned bone specimen and the diffracted beam was counted for 1200 s by an imaging plate (Fuji Film, Japan). From the obtained Debye ring (Fig. 2a), the diffracted intensity (*I*) from (002) and (310) planes were integrated along the azimuthal angle (*χ*) at an angle step of 1° and the intensity distributions as a function of *χ *(*I*(*χ*)) were approximated by the following function (modified ellipse) using a least-square method [36, 37]:

$$I(\chi )={\left\{\frac{{\mathrm{cos}}^{2}\left(\chi -\mu \right)}{{a}^{2}}-\frac{{\mathrm{sin}}^{2}\left(\chi -\mu \right)}{{b}^{2}}\right\}}^{-\frac{1}{2}}-c,$$

where *a*, *b*, *c*, and *μ* are fitting parameters and *μ* is the angle at which the intensity becomes maximum (Fig. 2b). Finally, the intensity ratio of (002)/(310) was calculated at each *χ* (Fig. 2c) in order to evaluate the degree of apatite orientation [16, 38]. This value for the randomly orientated apatite (NIST #2910: calcium hydroxyapatite) was confirmed to be 0.6. In the case of Fig. 2 which represents typical μXRD data from a rat femur middiaphysis, the apatite *c*-axis is predominantly oriented along the bone long axis, which is clear described with a radar diagram (Fig. 2d). The intensity ratio in the femur longitudinal axis was used for the assessment of the degree of preferential apatite *c*-axis orientation [13, 15].

### Statistical Analysis

All data are presented as mean ± standard deviation (SD) (*n* = 7 animals). The differences in *AR*, *DA,* and the degree of apatite *c*-axis orientation among the ROIs were analyzed using one-way ANOVA followed by Games-Howell multiple comparison test. Pearson’s correlation analysis was used to determine the relationships between *AR*, *DA* and the degree of apatite *c*-axis orientation. *P* < 0.05 was considered statistically significant. IBM SPSS Statistics Base 20 software for Windows (IBM, Japan) was used for all the statistical analyses.

## Results

### Osteocyte Canalicular Directionality Depending on Lacunar Elongation

**Fig. 3:**
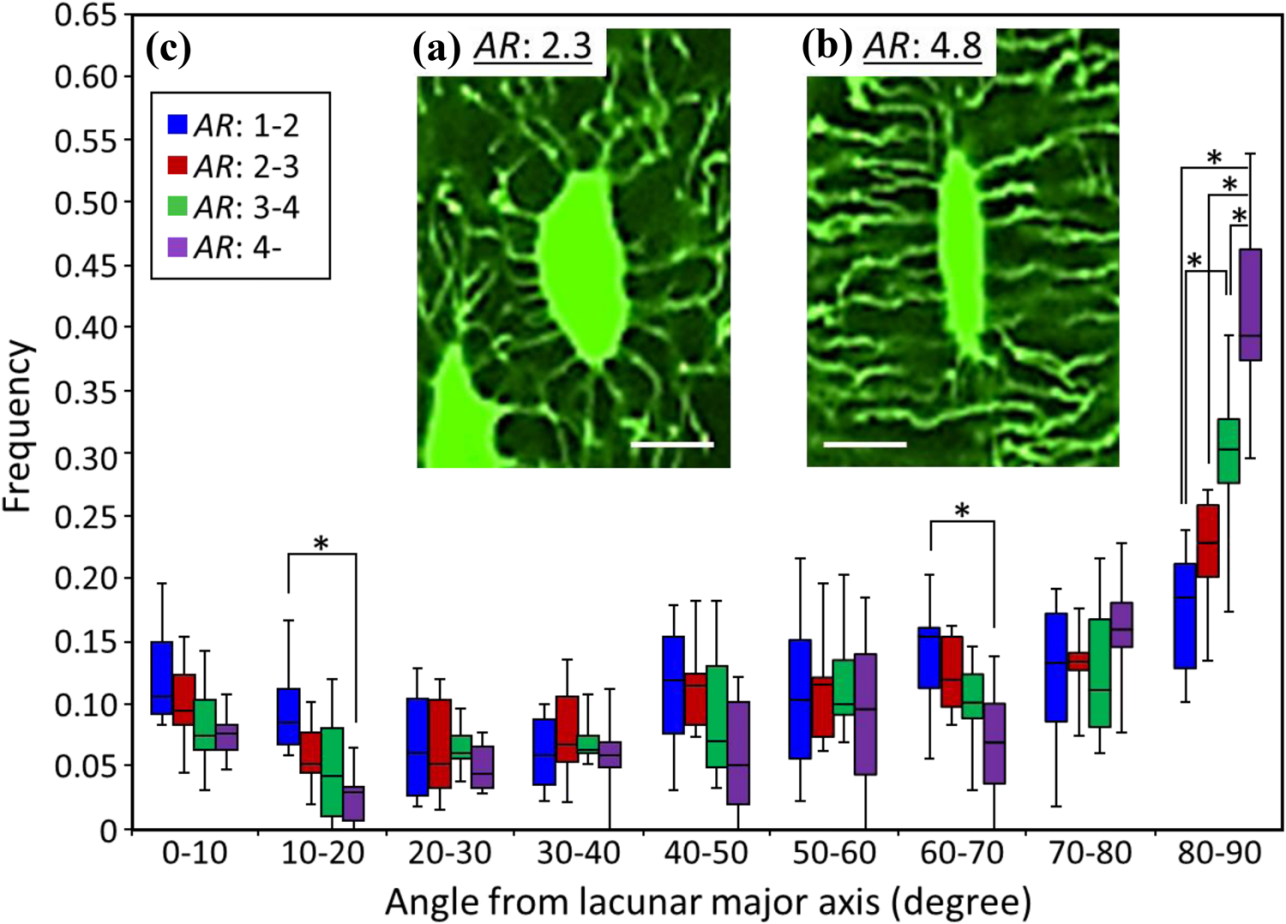
Osteocyte canalicular directionality distribution. **a**, **b** Representative confocal laser scanning microscopy images of the osteocyte lacunar-canalicular system acquired on a single z-plane. Scale bar: 10 μm. **c** Angular distribution of canaliculi that originated from the osteocyte lacuna with respect to the lacuna long axis, depending on the lacunar aspect ratio (*n* = 7). *Indicates statistical significance as determined by post hoc multiple comparison test (*P* < 0.05)

The osteocyte canaliculi originate in a radial fashion from the osteocyte lacuna, therefore, canalicular directionality seems to be largely affected by lacunar elongation as shown in Fig. 3a and b with relatively lower (2.3) and higher (4.8) *AR*, respectively. Figure 3c shows the frequency of canalicular direction, with respect to the lacunar long axis, as a function of lacunar *AR*. For relatively round osteocyte lacunae (*AR*: 1–2), the direction of distribution is almost homogeneous; the higher the lacunar *AR* is, the higher the frequency of canaliculi that ran perpendicularly to the lacunar long axis. For elongated lacunae with an aspect ratio > 4, more than 65% of canaliculi originated at 60° to 90° from the lacunar long axis. The directionality of the canaliculi is largely dependent on lacunar elongation.

### Morphology and Alignment of Osteocyte Lacunae Along with Femur Long Axis

**Fig. 4:**
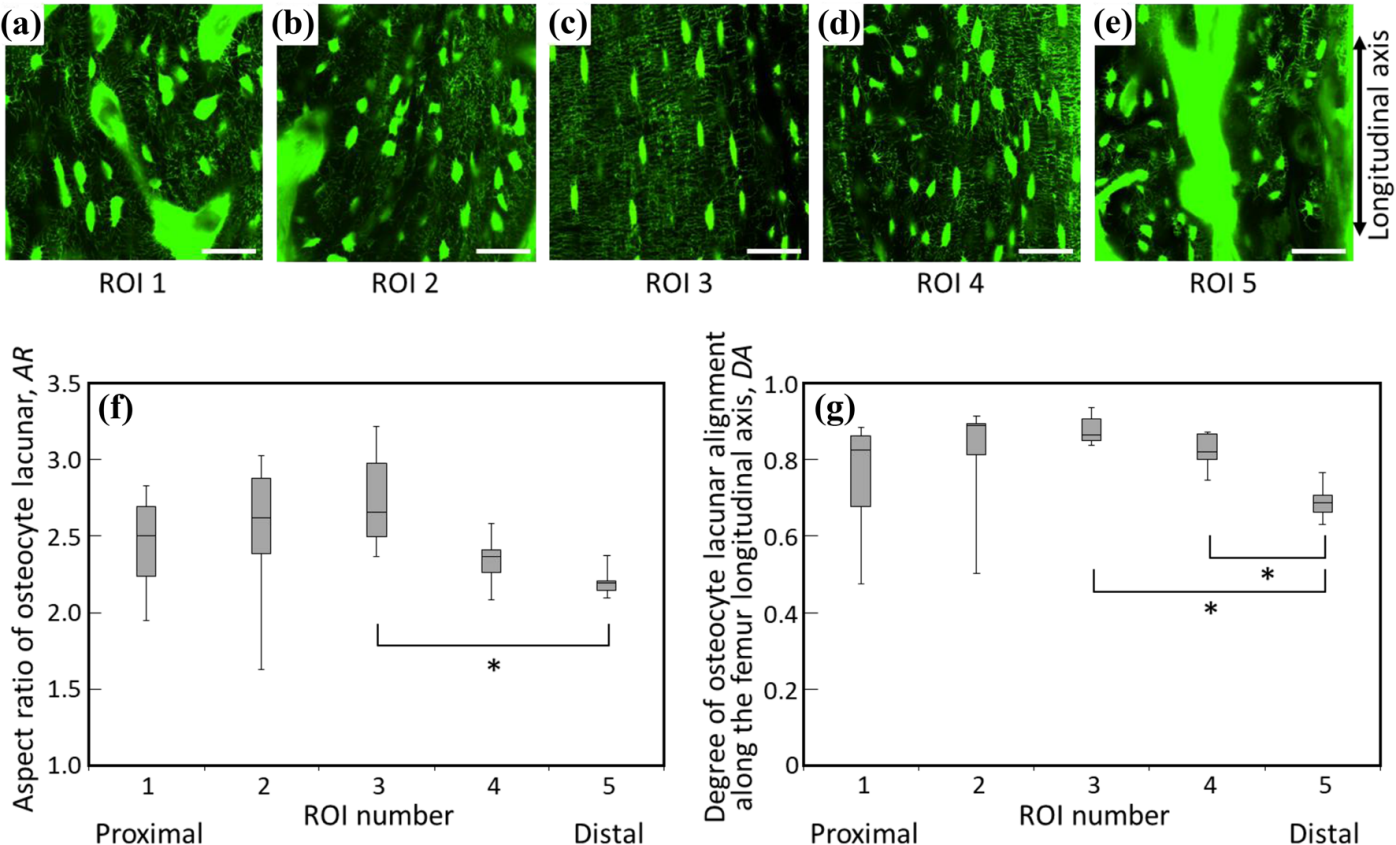
Observation and quantitative analyses of the osteocyte lacuno-canalicular system. **a**-**e** Confocal laser scanning microscopy images of the osteocyte lacuno-canalicular organization of each ROI. Scale bar: 50 μm. **f** Osteocyte lacunar aspect ratio (*AR*) of each ROI (*n* = 7). **g** The degree of the osteocyte lacunar alignment along the bone longitudinal axis (*DA*) of each ROI (*n* = 7). Both *AR* and *DA* showed a statistically significant position dependency (*P* < 0.05 for both) via a one-way ANOVA. *indicates statistical significance between ROIs determined by a post hoc multiple comparison test (*P* < 0.05). ROI, region of interest

Figure 4a-e shows typical FITC-stained images taken at each ROI. The *AR* and the degree of osteocyte lacunar alignment are quantified in Fig. 4f and g, respectively. Lacunae are highly elongated and their long axes are preferentially aligned in the femur longitudinal direction at the middiaphysis (ROI 3); they become less anisotropic in shape and show relatively dispersed direction of alignment in the proximal and distal cortices. Considering this and the canalicular directionality to the lacunar long axis, a large part of the canaliculi in the femur middiaphysis runs perpendicularly to the bone longitudinal direction.

### Preferential ECM Orientation Along with Femur Long Axis

**Fig. 5:**
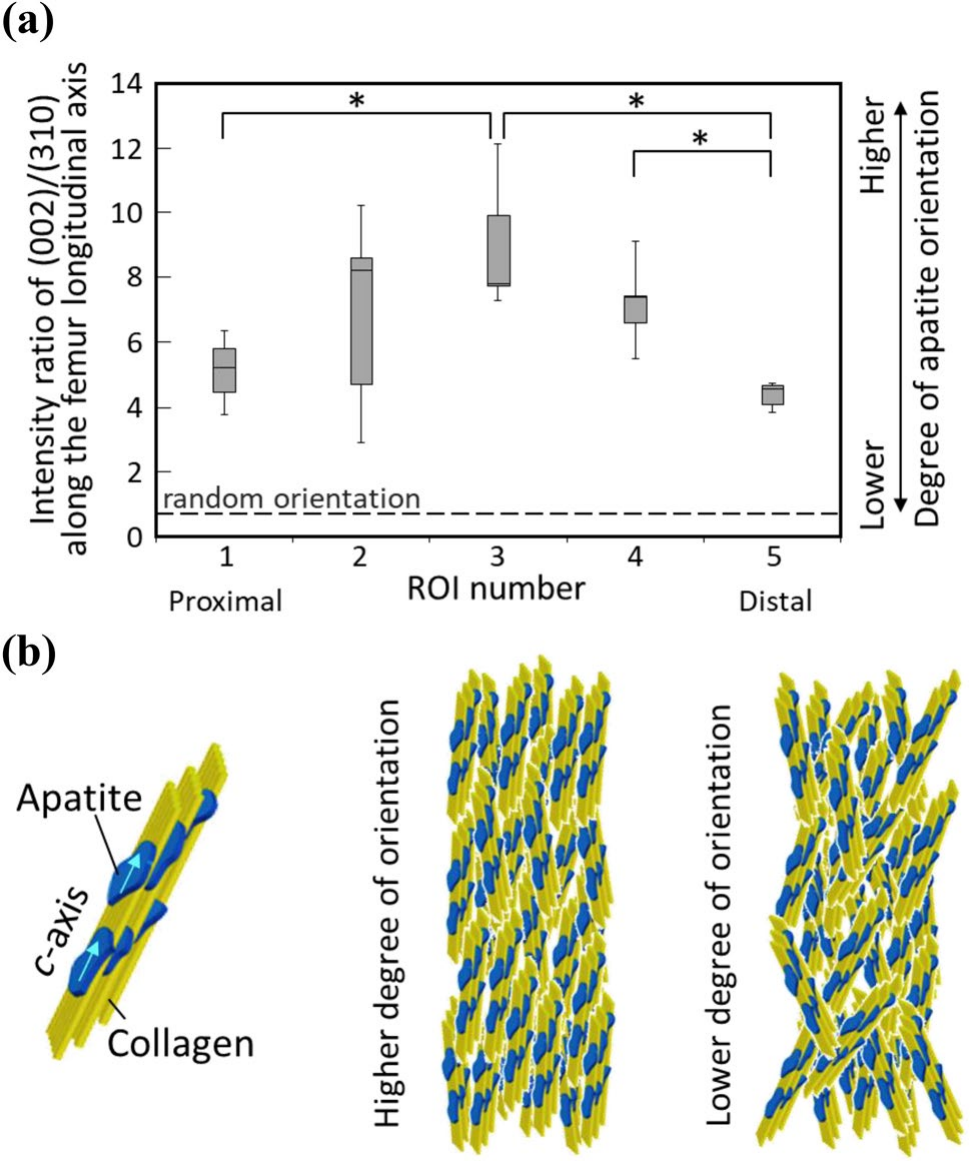
Analysis of ECM orientation. **a** Distribution of the degree of the preferential apatite *c*-axis orientation determined as the diffracted peak intensity ratio of (002)/(310) along the bone longitudinal axis (*n* = 7). The apatite orientation showed a statistically significant position dependency (*P* < 0.01) using a one-way ANOVA. *indicates statistical significance between ROIs determined by a post hoc multiple comparison test (*P* < 0.05). **b** Schematics of oriented apatite/collagen structure with higher and lower degree of orientation

The degree of the preferential apatite *c*-axis orientation calculated as the relative intensity ratio of (002) diffraction peak and (310) peak is shown in Fig. 5. In all ROIs, the intensity ratios along the femur longitudinal axis were over 0.6, showing that rat femur cortices show uniaxial preferential apatite *c*-axis orientation along the longitudinal axis in which the rat femur is predominantly loaded [29]. The degree of apatite orientation depends on the ROI (Fig. 2); it peaks at middiaphysis of the femur (ROI 3) and decreases in the proximal and distal ROIs.

### Correlations Between the Osteocyte and the Preferential ECM Orientation

**Fig. 6:**
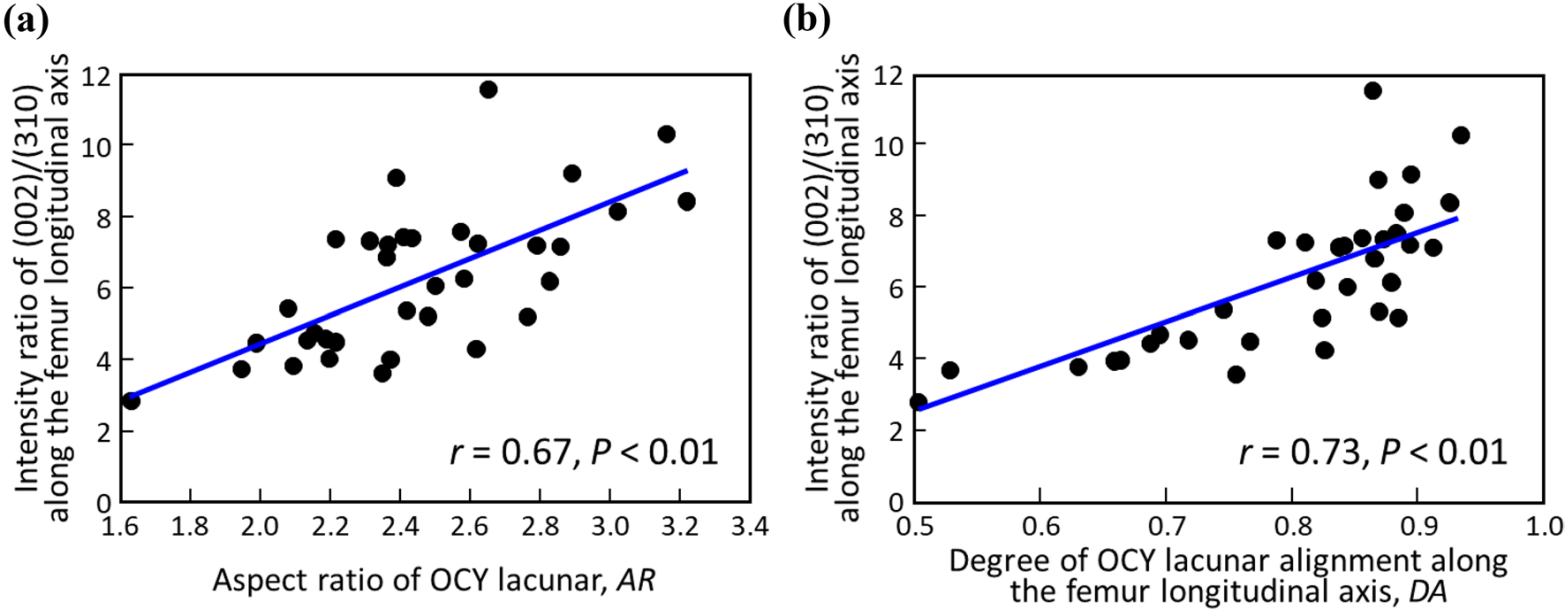
Anisotropic association between osteocyte architecture and ECM micro-organization. Correlations between the degree of the preferential apatite *c*-axis orientation and **a** the osteocyte lacunar aspect ratio (*r* = 0.67, *P* < 0.01), and **b** the degree of osteocyte lacunar alignment along the femur longitudinal axis (*r* = 0.73, *P* < 0.01). Thirty-five data sets (five ROIs for seven femurs) are plotted. ROI, region of interest

The degree of the apatite *c*-axis orientation was significantly and positively correlated with *AR* (*r* = 0.67, *P* < 0.01) and *DA* (*r* = 0.73, *P* < 0.01) of the osteocyte lacunae (Fig. 6). The degree of the apatite orientation synchronously varied with the osteocyte lacunar anisotropy along the femur longitudinal axis.

As a result, the bone portion with a higher degree of apatite *c*-axis orientation showed more osteocyte canaliculi perpendicular to the apatite oriented direction. Synchronous change of the degree of ECM orientation and that of osteocyte anisotropy is clearly recognized.

## Discussion

Anisotropic in vivo stress applied to bone should trigger the formation of anisotropic bone ECM organization because the bone needs to be strong enough to bear the anisotropic mechanical environment. The long bone is subjected to a mixture of axial load in the longitudinal axis and bending [29]. Bending is converted to axial stress along the longitudinal bone axis with a stress gradient from the endosteal to periosteal surface [39], hence, the long bone is predominantly loaded by a principal stress along its longitudinal axis. Moreover, during the direct measurement of bone strain using a strain gauge, the principal stress peaked at the long bone middiaphysis [40, 41]. As has been reported, the osteocyte in the uniaxially loaded bone elongates and aligns in the principally loaded direction. The osteocyte lacuna analyzed in the present study also anisotropically elongated and aligned with respect to the bone longitudinal axis (Fig. 4). The elongation and alignment of osteocytes peaked at the femur middiaphysis and became less anisotropic as a function of the distance from the middiaphysis. The directionality of canaliculi radiation from a lacuna is greatly influenced by the lacunar shape (Fig. 3). The more elongated lacunae possessed a higher frequency of canaliculi originating perpendicularly to the lacunar long axis. The directionality of the canaliculi is largely dependent on lacunar elongation. As a result, many of the canaliculi ran perpendicularly to the bone longitudinal axis at the middiaphysis, while the directionality became more random at the periphery. This supports the hypothesis proposed by Vatsa et al. [24] that the osteocyte optimizes its lacuno-canalicular distribution to adapt to the stress environment to exert moderate sensitivity as a mechanosensory cell. However, in the human osteonal bone, most of the canaliculi are aligned perpendicular to the osteonal lamellae; however, laterally extended canaliculi appear to co-align with collagen fibers within the lamellae [42], which is more complex than that in rat bone which lacks osteons.

This osteocyte lacunar-canalicular morphology should contribute to the effective detection of uniaxial stress. The theory based on fluid flow-induced shear stress stimulation around osteocyte was proposed as a most potent mechanosensing mechanism [43]. The osteocyte body and process which are located in a lacuna and canaliculus, respectively, are both considered to function as a stress (strain) sensing site [44, 45]; however, the in vitro study suggested that the mechanosensitivity of the osteocyte cell processes was much higher than that of the cell body [46]. When bone is loaded, the bone matrix surrounding the canalicular channels deforms and the canaliculus thin space is mechanically compressed, thereby, generating interstitial fluid flow in the canalicular channel which induces shear stress onto the cell membrane of the osteocyte process. The shear stress is the tangential force generated at the surface of the cell process by the gradient of velocity in the laminar fluid flow. Bones are subjected to different stress fields depending on the anatomical site, for example, the long bone is loaded by longitudinal stress [29]. Based on the fluid-flow theory, the directionality of the osteocyte canaliculi will affect the deformability of canalicular channels and subsequently, the efficiency of mechanosensing under the anisotropic stress field. However, the effect of osteocyte canaliculi directionality in mechanosensing has not been well documented to date.

**Fig. 7:**
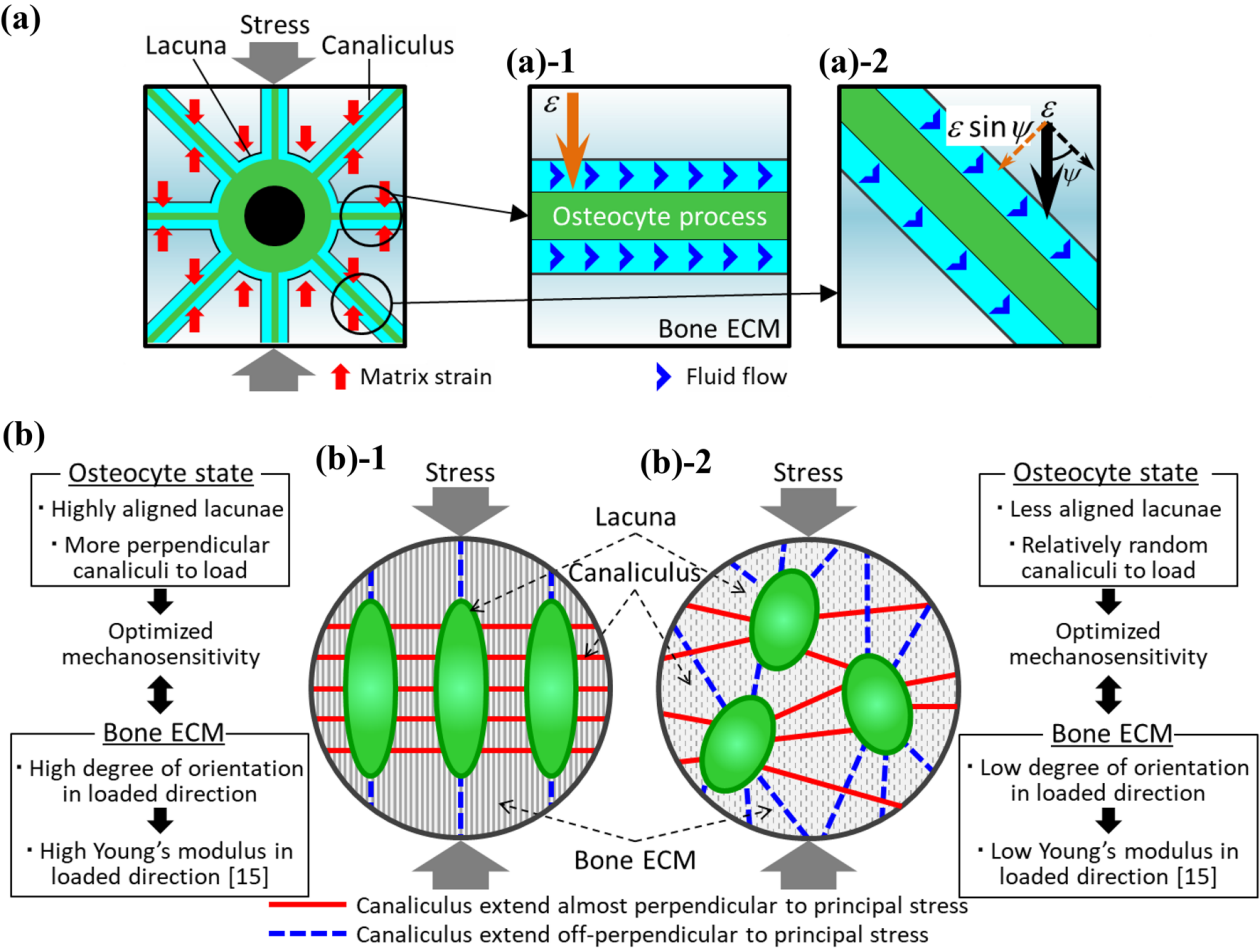
Possible relation between octeocytic lacuno-canalicular architecture and mechanosensation and ECM orientation. **a** Schematic representation of how the osteocyte’s mechanosensitivity is affected by the canalicular direction to the principal load based on the fluid-flow theory regarding a single canaliculus. **b** Schematic comparison of mechanosensitivity and resultant formation of preferential apatite orientation between the two types of osteocyte lacuno-canalicular organizations: elodngated and highly aligned lacunae with homogeneous directionality of their canaliculi perpendicular to the principal load, and randomly distributed lacunae with scattered canalicular directionality. *ε* indicates matrix strain

In Fig. 7a, in a canaliculus extended perpendicularly to the principal stress direction, the entire part of tissue strain (1*ε*) is used to deform the canaliculus, resulting in the effective generation of fluid flow in the canaliculus. In contrast, in a canaliculus extended obliquely to the stress direction, only a part of tissue strain (*ε *sin*ψ*) was used to deform the canaliculus as a function of inclination angle *ψ,* which might mean that in the obliquely extended canaliculus, the interstitial fluid-flow rate was smaller than in the perpendicularly extended canaliculus to the principal stress direction. The magnitude of shear stress is dominated by the fluid-flow rate [47, 48] and bone cell response to fluid shear stress is fluid-flow rate-dependent. Bakker et al. [48] reported that the production of NO and PGE2 from bone cells which regulate osteoclastic and osteoblastic activities was enhanced in a shear stress rate-dependent manner. Bacabac et al. [49] found a similar fluid shear stress rate dependency of NO production. According to an analytical model, the fluid-flow rate varied among canaliculi depending on the canalicular radiation direction [50]. As illustrated in Fig. 7b, an osteocyte that is elongated and aligned in the loading direction, with increased unidirectionality of its canaliculi and processes, may show enhanced sensitivity to the specific stress field–the uniaxial principal stress that is perpendicular to the canaliculi. Moreover, a lacuna that is elongated and aligned in the loading direction experiences less strain (deformation) than one that is aligned in the off-loading direction or is spherically shaped. Having lacunae that are parallel to the loading direction would be beneficial for osteocyte mechanosensing because the fluid flow generated by canalicular deformation can be sensed without being disturbed by deformation of the lacuna. In contrast, the osteocyte with canaliculi which randomly extend is unsuitable for sensing specific uniaxial principal stress; conversely, such osteocytes, typically seen in the non-weight-bearing bone [51–53], are considered more sensitive to weak and isotropic stresses [53].

The degree of preferential *c*-axis orientation of apatite significantly correlated with osteocyte anisotropy (Fig. 6), which leads to the hypothesis that the osteocyte regulates the production of oriented ECM in response to the magnitude of applied stress the osteocyte detects, although this production mechanism is largely unclear. Kerschnitzki et al. [54] proposed that the aligned and collective action of ECM-producing osteoblasts, which might be regulated by signaling from osteocytes, is required for the formation of a highly oriented ECM. In vitro investigation by Matsugaki et al. [55] showed the aligned osteoblast forms oriented ECM whose degree is dependent on the degree of osteoblast alignment, which partly supports the hypothesis of Kerschnitzki et al. Further studies should be performed to clarify how the osteocyte translates the information from the anisotropic stress field into the factors that regulate the formation of the ECM orientation, focusing on the cellular behaviors and production and distribution of soluble agents induced by mechanical stimulation to govern cellular behaviors.

The relationships between the magnitude of stress, osteocyte anisotropy, and ECM orientation will provide important insight into the functional adaptation of the bone. As has been discussed thus far, adaptive responses of bone to mechanical environment take place through changes in bone mass or bone geometry [56] which is an important index contributing to the overall mechanical functions of bone from the structural aspect. However, the adaptive response of the intrinsic material parameters, that is, the other essential aspects of the mechanical functions independent of the structural parameters, remains poorly understood. Material anisotropy, as represented by the ECM orientation in this study, might be an important material parameter because it can strengthen the bone in the dedicated direction where the principal load is applied, which effectively optimizes the mechanical function of the bone under anisotropic mechanical conditions. On the contrary, changing bone quantity inevitably increases bone strength in not only principally loaded direction but also in other all directions, which may not always be efficient.

## Conclusion

The osteocyte lacuno-canalicular network and preferential ECM orientation in male rat femur were quantitatively analyzed to understand their correlation. We first demonstrated the quantitative correlation between the anisotropy in the osteocyte lacuno-canalicular network and that in the ECM arrangement. Osteocyte canaliculi that take the central role in mechanosensing originated radially from the lacuna; therefore, canalicular directionality largely depended on lacunar elongation and alignment. Bone portions including osteocytes with highly anisotropic lacuno-canalicular system with respect to the bone longitudinal axis showed a highly oriented bone ECM to enhance Young’s modulus along the loaded direction. Modification of ECM anisotropy may be able to strengthen bone in the loaded direction more efficiently, which is very interesting as a new aspect of bone function adaptation.

## References

[1] Dallas SL, Moore DS (2020). Using confocal imaging approaches to understand the structure and function of osteocytes and the lacunocanalicular network. Bone.

[2] Bellido T (2014). Osteocyte-driven bone remodeling. Calcif Tissue Int.

[3] Klein-Nulend J, Bakker AD (2007). Osteocytes: mechanosensors of bone and orchestrators of mechanical adaptation. Clin Rev Bone Miner Metab.

[4] Burger EH, Klein-Nulend J (1999). Mechanotransduction in bone–role of the lacuno-canalicular network. FASEB J.

[5] Carina V, Bella ED, Costa V, Bellavia D, Veronesi F, Cepollaro S, Fini M, Giavaresi G (2020). Bone’s response to mechanical loading in aging and osteoporosis: molecular mechanisms. Calcif Tissue Int.

[6] Cabahug-Zuckerman P, Frikha-Benayed D, Majeska RJ, Tuthill A, Yakar S, Judex S, Schaffler MB (2016). Osteocyte apoptosis caused by hindlimb unloading is required to trigger osteocyte RANKL production and subsequent resorption of cortical and trabecular bone in mice femurs. J Bone Miner Res.

[7] Metzger CE, Brezicha JE, Elizondo JP, Narayan SA, Hogan HA, Bloomfield SA (2017). Differential responses of mechanosensitive osteocyte proteins in fore- and hindlimbs of hindlimb-unloaded rats. Bone.

[8] Tajima T, Menuki K, Okuma KF, Tsukamoto M, Fukuda H, Okada Y, Kosugi K, Yamanaka Y, Uchida S, Sakai A (2018). Cortical bone loss due to skeletal unloading in aldehyde dehydrogenase 2 gene knockout mice is associated with decreased PTH receptor expression in osteocytes. Bone.

[9] Hemmatian H, Jalali R, Semeins CM, Hogervorst JMA, van Lenthe GH, Klein-Nulend J, Bakker AD (2018). Mechanical loading differentially affects osteocytes in fibulae from lactating mice compared to osteocytes in virgin mice: possible role for lacuna size. Calcif Tissue Int.

[10] Lara-Castillo N, Kim-Weroha NA, Kamel MA, Javaheri B, Ellies DL, Krumlauf RE, Thiagarajan G, Johnson ML (2015). In vivo mechanical loading rapidly activates β-catenin signaling in osteocytes through a prostaglandin mediated mechanism. Bone.

[11] Bach-Gansmo FL, Wittig NK, Brüel A, Thomsen JS, Birkedal H (2016). Immobilization and long-term recovery results in large changes in bone structure and strength but no corresponding aterations of osteocyte lacunar properties. Bone.

[12] Yang J, Li J, Cui X, Li W, Xue Y, Shang P, Zhang H (2020). Blocking glucocorticoid signaling in osteoblasts and osteocytes prevents mechanical unloading-induced cortical bone loss. Bone.

[13] Sekita A, Matsugaki A, Ishimoto T, Nakano T (2017). Synchronous disruption of anisotropic arrangement of the osteocyte network and collagen/apatite in melanoma bone metastasis. J Struct Biol.

[14] Silva MJ, Brodt MD, Wopenka B, Thomopoulos S, Williams D, Wassen MH, Ko M, Kusano N, Bank RA (2006). Decreased collagen organization and content are associated with reduced strength of demineralized and intact bone in the SAMP6 mouse. J Bone Miner Res.

[15] Ishimoto T, Nakano T, Umakoshi Y, Ymamoto M, Tabata Y (2013). Degree of biological apatite c-axis orientation rather than bone mineral density controls mechanical function in bone regenerated using recombinant bone morphogenetic protein-2. J Bone Miner Res.

[16] Nakano T, Kaibara K, Tabata Y, Nagata N, Enomoto S, Marukawa E, Umakoshi Y (2002). Unique alignment and texture of biological apatite crystallites in typical calcified tissues analyzed by microbeam X-ray diffractometer system. Bone.

[17] Li S, Demirci E, Silberschmid VV (2013). Variability and anisotropy of mechanical behavior of cortical bone in tension and compression. J Mech Behav Biomed Mater.

[18] Katz JL (1980). Anisotropy of Young’s modulus of bone. Nature.

[19] Shinno T, Ishimoto T, Saito M, Uemura R, Arino M, Marumo K, Nakano T, Hayashi M (2016). Comprehensive analyses of how tubule occlusion and advanced glycation end-products diminish strength of aged dentin. Sci Rep.

[20] Peterlik H, Roschger P, Klaushofer K, Fratzl P (2012). From brittle to ductile fracture of bone. Nature Mater.

[21] Nakano T, Kaibara K, Ishimoto T, Tabata Y, Umakoshi Y (2012). Biological apatite (BAp) crystallographic orientation and texture as a new index for assessing the microstructure and function of bone regenerated by tissue engineering. Bone.

[22] Liu Y, Manjubala I, Schell H, Epari DR, Roschger P, Duda GN, Fratzl P (2010). Size and habit of mineral particles in bone and mineralized callus during bone healing in sheep. J Bone Miner Res.

[23] Ishimoto T, Nakano T, Yamamoto M, Tabata Y (2011). Biomechanical evaluation of regenerated long bone by nanoindentation. J Mater Sci Mater Med.

[24] Vatsa A, Semeins CM, Smit TH, Klein-Nulend J (2008). Paxillin localisation in osteocytes—Is it determined by the direction of loading?. Biochem Biophys Res Commun.

[25] Sawada Y, Sheetz MP (2002). Force transduction by triton cytoskeletons. J Cell Biol.

[26] Wang N, Butler JP, Ingber DE (1993). Mechanotransduction across the cell-surface and through the cytoskeleton. Science.

[27] Maniotis AJ, Chen CS, Ingber DE (1997). Demonstration of mechanical connections between integrins, cytoskeletal filaments, and nucleoplasm that stabilize nuclear structure. Proc Natl Acad Sci USA.

[28] Klein-Nulend J, Bacabac RG, Bakker D (2012). Mechanical loading and how it affects bone cells: the role of the osteocyte cytoskeleton in maintaining our skeleton. Eur Cell Mater.

[29] Wehner T, Wolfram U, Henzler T, Niemeyer F, Claes L, Simon U (2010). Internal forces and moments in the femur of the rat during gait. J Biomech.

[30] Ciani C, Doty SD, Fritton SP (2009). An effective histological staining process to visualize bone interstitial fluid space using confocal microscopy. Bone.

[31] Kamioka H, Honjo T, Takano-Yamamoto T (2001). A three-dimensional distribution of osteocyte processes revealed by the combination of confocal laser scanning microscopy and differential interference contrast microscopy. Bone.

[32] Carpenter AE, Jones TR, Lamprecht MR, Clarke C, Kang IH, Friman O, Guertin DA, Chang JH, Lindquist RA, Moffat J, Golland P, Sabatini DM (2006). Cell Profiler: image analysis software for identifying and quantifying cell phenotypes. Genome Biol.

[33] Nakanishi Y, Matsugaki A, Kawahara K, Ninomiya T, Sawada H, Nakano T (2019). Unique arrangement of bone matrix orthogonal to osteoblast alignment controlled by Tspan11-mediated focal adhesion assembly. Biomaterials.

[34] Landis WJ (1995). The strength of a calcified tissue depends in part on the molecular structure and organization of its constituent mineral crystals in their organic matrix. Bone.

[35] Ozasa R, Ishimoto T, Miyabe S, Hashimoto J, Hirao M, Yoshikawa H, Nakano T (2019). Osteoporosis changes collagen/apatite orientation and Young’s modulus in vertebral cortical bone of rat. Calcif Tissue Int.

[36] Noyama Y, Nakano T, Ishimoto T, Sakai T, Yoshikawa H (2013). Design and optimization of the oriented groove on the hip implant surface to promote bone microstructure integrity. Bone.

[37] Kuroshima S, Nakano T, Ishimoto T, Sasaki M, Inoue M, Yasutake M, Sawase T (2017). Optimally oriented grooves on dental implants improve bone quality around implants under repetitive mechanical loading. Acta Biomater.

[38] Ishimoto T, Yamada K, Takahashi H, Takahata M, Ito M, Hanawa T, Nakano T (2018). Trabecular health of vertebrae based on anisotropy in trabecular architecture and collagen/apatite micro-arrangement after implantation of intervertebral fusion cages in the sheep spine. Bone.

[39] Kameo Y, Adachi T (2014). Interstitial fluid flow in canaliculi as a mechanical stimulus for cancellous bone remodeling: in silico validation. Biomech Model Mechanobiol.

[40] Biewener AA, Taylor CR (1986). Bone strain: a determinant of gait and speed?. J Exp Biol.

[41] Biewener AA (1983). Allometry of quadrupedal locomotion: the scaling of duty factor, bone curvature and limb orientation to body size. J Exp Biol.

[42] Repp F, Kollmannsberger P, Roschger A, Berzlanovich A, Gruber GM, Roschger P, Wagermaier W, Weinkamer R (2017). Coalignment of osteocyte canaliculi and collagen fibers in human osteonal bone. J Struct Biol.

[43] Weinbaum S, Cowin SC, Zeng Y (1994). A model for the excitation of osteocytes by mechanical loading-induced bone fluid shear stress. J Biomech.

[44] Klein-Nulend J, Bakker AD, Bacabac RG, Vatsa A, Weinbaum S (2013). Mechanosensation and transduction in osteocytes. Bone.

[45] Vatsa A, Mizuno D, Smit TM, Schmidt CF, MacKintosh FC, Klein-Nulend J (2006). Bio imaging of intracellular NO production in single bone cells after mechanical stimulation. J Bone Miner Res.

[46] Adachi T, Aonuma Y, Tanaka M, Hojo M, Takano-Yamamoto T, Kamioka H (2009). Calcium response in single osteocytes to locally applied mechanical stimulus: differences in cell process and cell body. J Biomech.

[47] Jia Y, Bagnaninchi PO, Yang Y, El Haj AJ, Hinds MT, Kirkpatrick SJ, Wang R (2009). Doppler optical coherence tomography imaging of local fluid flow and shear stress within microporous scaffolds. J Biomed Opt.

[48] Bakker AD, Soejima K, Klein-Nulend J, Burger EH (2001). The production of nitricoxide and prostaglandin E2 by primary bone cells is shear stress dependent. J Biomech.

[49] Bacabac RG, Smit TH, Mullender MG, Dijcks SJ, Van Loon JJ, Klein-Nulend J (2014). Nitric oxide production by bone cells is fluid shear stress rate dependent. Biochem Biophys Res Commun.

[50] Srinivasan S, Gross TS (2000). Canalicular fluid flow induced by bending of a long bone. Med Eng Phys.

[51] Vatsa A, Breuls RG, Semeins CM, Salmon PL, Smit TH, Klein-Nulend J (2008). Osteocyte morphology in fibula and calvaria—Is there a role for mechanosensing?. Bone.

[52] Sugawara Y, Kamioka H, Ishihara Y, Fujisawa N, Kawanabe N, Yamashiro T (2013). The early mouse 3D osteocyte network in the presence and absence of mechanical loading. Bone.

[53] Wang J, Ishimoto T, Nakano T (2017). Unloading-induced degradation of the anisotropic arrangement of collagen/apatite in rat femurs. Calcif Tissue Int.

[54] Kerschnitzki M, Wagermaier W, Roschger P, Seto J, Shahar R, Duda GN, Mundlos S, Fratzl P (2011). The organization of the osteocyte network mirrors the extracellular matrix orientation in bone. J Struct Biol.

[55] Matsugaki A, Isobe Y, Saku T, Nakano T (2015). Quantitative regulation of bone-mimetic, oriented collagen/apatite matrix structure depends on the degree of osteoblast alignment on oriented collagen substrates. J Biomed Mater Res A.

[56] Meakin LB, Price JS, Lanyon LE (2014). The contribution of experimental in vivo models to understanding the mechanisms of adaptation to mechanical loading in bone. Front Endocrinol.

